# Supplementary material for: The Emergency Performance of the Hungarian Ambulance Service during the COVID-19 Pandemic
Source: Healthcare (Basel). 2022 Nov 21;10(11):2331. doi: 10.3390/healthcare10112331 (PMC9690681; doi:10.3390/healthcare10112331)
Supplement: Supplementary file 1 [file healthcare-10-02331-s001.zip › healthcare-1953078-supplementary.pdf]

Table S1. Number and distribution of various non-COVID-19 related deliveries by the NAS between 2019 and 2021

	2019		2020		2021		2019/2020	2019/2021	2020/2021
	N	%	N	%	N	%	p-value	p-value	p-value
**Acute myocardial infarction (ICD10: I21, I22)**									
**Gender**									
male	1,787	56.4%	2,024	56.9%	2,030	57.4%	0.531	0.553	0.600
female	1,074	33.8%	1,232	34.6%	1,203	34.0%			
missing	312	9.8%	302	8.5%	304	8.6%			
**Age**									
15-64	1,611	50.8%	1,767	49.7%	1,798	50.9%	0.366	0.968	0.332
65+	1,535	48.3%	1,760	49.5%	1,710	48.3%			
missing	27	0.9%	31	0.8%	29	0.8%			
**Haemorrhagic stroke (ICD10: I60, I61, I62)**									
**Gender**									
male	155	42.7%	110	45.7%	98	44.4%	0.537	0.744	0.801
female	158	43.5%	109	45.2%	98	44.3%			
missing	50	13.8%	22	9.1%	25	11.3%			
**Age**									
15-64	196	54.0%	111	46.1%	119	53.8%	0.045*	0.738	0.120
65+	159	43.8%	126	52.3%	101	45.7%			
missing	8	2.2%	4	1.6%	1	0.5%			
**Ischemic stroke (ICD10: I63, I64)**									
**Gender**									
male	8,888	44.3%	9,630	44.7%	10,225	45.2%	0.379	0.152	0.264
female	9,221	45.9%	9,811	45.6%	10,423	46.1%			
missing	1,961	9.8%	2,095	9.7%	1,971	8.7%			
**Age**									
15-64	5,692	28.4%	5,940	27.6%	6,189	27.4%	0.064	0.016*	0.581
65+	14,279	71.1%	15,516	72.0%	16,357	72.3%			
missing	99	0.5%	80	0.4%	73	0.3%			
**Stroke (ICD10: I60, I61, I62, I63, I64)**									
**Gender**									
male	9,043	44.3%	9,740	44.7%	10,323	45.2%	0.371	0.139	0.276
female	9,379	45.9%	9,920	45.6%	10,521	46.1%			
missing	2,011	9.8%	2,117	9.7%	1,996	8.7%			
**Age**									
15-64	5,888	28.4%	6,051	27.8%	6,308	27.6%	0.015*	0.004*	0.659
65+	14,438	71.1%	15,642	71.8%	16,458	72.1%			
missing	107	0.5%	84	0.4%	74	0.3%			
**Overall ambulance deliveries (without COVID-19)**									
**Gender**									
male	316,618	38.7%	327,081	38.9%	372,144	39.2%	0.009*	<0.001*	<0.001*
female	332,182	40.6%	340,076	40.4%	393,422	41.5%			
missing	169,057	20.7%	174,270	20.7%	182,764	19.3%			
**Age**									
15-64	424,215	51.9%	430,253	51.2%	482,786	50.9%	<0.001*	<0.001*	<0.001*
65+	350,751	42.9%	367,252	43.6%	424,655	44.8%			
missing	42,891	5.2%	43,922	5.2%	40,889	4.3%			

*Significant ($p < 0.05$). Note: The years in this table show the data from 1st January to 31st December, not from week 1 to week 52.
